# A Hospital-Based Quality Improvement Initiative to Reduce Postdural Puncture Headache in Cesarean Deliveries

**DOI:** 10.7759/cureus.79159

**Published:** 2025-02-17

**Authors:** Fadlalraham Abdelrahim Hassan, Ali Hadi M Alhajri, Mohammed Shoaib, Nisrin Magboul Elfadel Magboul, Nedra Naser Akremi Hammedi, Larcy Nice, Margie Asistin, Vanessa Demata

**Affiliations:** 1 Anesthesiology, Najran Armed Forces Hospital, Ministry of Defense Healthcare Services, Najran, SAU; 2 Medical Administration, Najran Armed Forces Hospital, Ministry of Defense Healthcare Services, Najran, SAU; 3 Obstetrics and Gynaecology, Najran Armed Forces Hospital, Ministry of Defense Healthcare Services, Najran, SAU; 4 Operating Room, Najran Armed Forces Hospital, Ministry of Defense Healthcare Services, Najran, SAU; 5 Infectious Diseases, Najran Armed Forces Hospital, Ministry of Defense Healthcare Services, Najran, SAU

**Keywords:** anesthesia management, cesarean delivery, epidural blood patch, post-dural puncture headache (pdph), quality improvement

## Abstract

Background: Postdural puncture headache (PDPH) is a major complication of neuraxial anesthesia in cesarean deliveries. This study aimed to reduce PDPH incidence through a quality improvement initiative focusing on operating room (OR) efficiency and anesthetic management.

Methods: Using Plan-Do-Study-Act (PDSA) cycles, interventions included preoperative catheterization, synchronized scrubbing, and improved surgeon-anesthetist coordination. Spinal anesthesia protocols were refined with atraumatic needle techniques and standardized vasopressor use. Data from the pre-intervention (April to October 2023) and post-intervention (November 2023 to April 2024) periods were compared.

Results: OR time decreased from 105 to 85 minutes. PDPH incidence dropped from 16% (21/140 lower segment cesarean sections (LSCS)) to 1.5% (2/127 LSCS). Vasopressor use declined, with ephedrine doses reducing from 17 mg to 6 mg and phenylephrine from 30 mcg to 5 mcg, improving hemodynamic stability.

Conclusion: Workflow enhancements effectively reduced PDPH incidence by optimizing OR efficiency and anesthetic management. Iterative PDSA cycles and real-time feedback contributed to sustained improvements, offering a scalable model for cesarean delivery quality improvement.

## Introduction

Postdural puncture headache (PDPH), a well-documented complication of neuraxial anesthesia, poses significant challenges in obstetric care, often prolonging hospital stays, increasing healthcare costs, and adversely affecting maternal recovery [1]. Despite advances in spinal anesthesia techniques, PDPH incidence rates remain variable, with studies reporting frequencies as high as 10-20% in some settings [2]. This variability underscores the need for standardized, evidence-based protocols to mitigate risks while maintaining procedural efficiency [3]. At the heart of this challenge lies the interplay between operating room (OR) efficiency, anesthetic practices, and hemodynamic stability. Prolonged OR times have been implicated in increased maternal exposure to positional changes, fluid shifts, and prolonged spinal anesthesia effects, which are all potential contributors to PDPH [4]. Concurrently, extended surgical durations often correlate with higher vasopressor requirements to counteract spinal-induced hypotension, further complicating perioperative management [5].

In response to these challenges, this quality improvement project addressed those factors in OR workflows and anesthetic management that were modifiable to reduce the incidence of PDPH in cesarean deliveries. The project was built on the foundation of streamlining OR processes to reduce OR stay, thereby minimizing maternal exposure to spinal anesthesia-related complications and improving hemodynamic stability. Phase interventions, including preoperative catheterization, synchronized team scrubbing, and optimized surgeon-anesthetist coordination, were implemented using Plan-Do-Study-Act (PDSA) cycles in a multidisciplinary team [4]. They were further supported by adherence to an updated spinal anesthesia protocol emphasizing atraumatic needle techniques and standardized administration of vasopressors [6]. To accomplish this, the design of the initiative included both quantitative metrics (OR time, PDPH rates, and vasopressor consumption) and qualitative clinical and frontline staff input to refine real-time processes. It controlled for variability in the patient population by focusing only on elective and emergency cesarean cases and only on their impact on clinical outcomes [7, 8].

The significance of this initiative lies in its dual focus on operational efficiency and patient-centered outcomes. While prior studies have examined isolated strategies for PDPH reduction, this project uniquely demonstrates how systemic OR workflow enhancements can synergistically reduce both procedural time and anesthesia-related complications. The observed correlation between shorter OR durations and decreased vasopressor requirements provides novel insights into the hemodynamic benefits of efficient surgical practices [9]. Furthermore, the integration of weekly team feedback into iterative PDSA cycles highlights the value of frontline clinician engagement in sustaining process improvements. As healthcare systems increasingly prioritize value-based care, this initiative offers a replicable model for balancing clinical excellence with operational efficiency in obstetric anesthesia. By addressing the multifactorial contributors to PDPH through coordinated process redesign, the project advances the understanding of how system-level interventions can directly enhance patient safety and resource utilization in high-volume surgical settings.

## Materials and methods

This study was conducted at Najran Armed Forces Hospital, Saudi Arabia, a multidisciplinary medical facility specializing in obstetric care, located in Najran. The recruitment period spanned from April 2023 to April 2024, covering both the pre-intervention (April 2023 to October 2023) and intervention (November 2023 to April 2024) phases. Patients undergoing lower segment cesarean section (LSCS) were included in the study. The inclusion criteria comprised all elective and emergency LSCS cases performed under spinal anesthesia. Patients were excluded if they had contraindications to spinal anesthesia, incomplete medical records, or underwent LSCS with general anesthesia. A consecutive sampling method was employed, ensuring that all eligible LSCS cases during the study period were included for analysis.

Quantitative analysis

The primary quantitative method used was descriptive statistics to summarize key variables such as OR time, PDPH incidence, and vasopressor use. Mean OR times were calculated for both the pre-intervention and post-intervention periods. The incidence of PDPH was calculated as a percentage of total LSCS cases in each period (pre- and post-intervention).

Pre-intervention

A total of 140 LSCS procedures were performed, with an average OR time of 105 minutes and a PDPH incidence of 16% (21 cases).

Post-intervention

A total of 127 LSCS procedures were performed, with an average OR time of 85 minutes and a PDPH incidence of 1.5% (2 cases).

Comparative Analysis

Pre- and post-intervention data were compared to assess the impact of the intervention on OR time, PDPH incidence, and vasopressor use. The percentage reduction in PDPH cases and the percentage decrease in OR time were key outcome measures.

Vasopressor Use

During the observation period, higher consumption of vasopressors was observed, with approximately 17 doses of ephedrine (6 mg per dose) and 30 doses of phenylephrine (100 mcg per dose) administered per procedure. Following the implementation of the intervention, the project period showed a marked reduction in vasopressor use, with both ephedrine and phenylephrine consumption decreasing significantly (approximately 6 doses of ephedrine and 5 doses of phenylephrine).

Inference

The significant reduction in vasopressor use can be directly linked to the reduction in OR time. As OR time decreased from 105 minutes to 85 minutes on average, patient hemodynamic stability improved, thereby reducing the need for vasopressor intervention to manage intraoperative hypotension.

Qualitative analysis

Qualitative data were gathered informally through weekly feedback from the surgical and anesthesia teams. This feedback focused on perceived challenges and successes of the intervention, including team coordination and ease of adherence to the new procedural steps. These data were used to make real-time adjustments during the PDSA cycles, ensuring continuous improvement in the intervention's implementation.

Methods for Understanding Variation: Time-Series Analysis

OR time: A time-series analysis was employed to track changes in OR time across the intervention period. Weekly OR times were recorded and averaged, allowing the team to observe trends and variations in OR time as the intervention progressed. The goal was to assess how quickly OR times decreased following the implementation of specific changes, which were introduced sequentially across different PDSA cycles but executed simultaneously within each surgical procedure (e.g., simultaneous scrubbing of the anesthetist and surgeon, and early catheter insertion before patient transfer to the OR).

PDPH incidence: PDPH incidence was tracked over time to determine whether reductions in OR time corresponded to a decrease in PDPH cases. By reviewing cases weekly, the team could monitor if improvements in OR efficiency led to fewer PDPH cases, supporting the hypothesis that reduced OR time would lower the risk of this complication.

Control of Confounding Variables

Consistency in patient population: Only elective LSCS cases or only emergency LSCS cases should have been included in the analysis to ensure uniformity and control for patient variability. However, both groups were analyzed together while maintaining consistency in other variables, such as anesthesia type and procedural protocol, to minimize potential confounding factors. This consistency ensured that variations in PDPH incidence and OR time were attributable to the intervention rather than differences in patient health status or procedure type.

Adjustments during PDSA cycles: As variations in OR time or complications were identified, real-time adjustments were made through the PDSA cycles. For example, if a particular step in the process (e.g., surgeon arrival time) was found to be inconsistent, corrective actions were taken in the next cycle. This allowed the team to understand and address variations as they arose, ensuring that time-related improvements were sustained.

Ethical considerations

This study was approved by the Research Ethics Committee (REC) of the Academic Affairs and Training Department, Najran Armed Forces Hospital, Saudi Arabia (Code: NAFHMREC/2023/AN/1). Patient data were collected retrospectively and prospectively from medical records, with all information handled through the hospital’s electronic medical records (EMR) system in compliance with data privacy regulations. No identifiable patient information was shared outside the clinical team, ensuring confidentiality. There were no conflicts of interest, as all project members were hospital employees, and no external organizations were involved in the design, implementation, or analysis of the project.

## Results

The intervention began with the implementation of the PDSA cycles, which evolved over three distinct phases:

PDSA Cycle 1 (November 2023)

The team focused on reducing OR time by implementing procedural changes, such as early insertion of urinary catheters in the ward, calling the surgeon and patient simultaneously to the OR, and having the anesthesia technician prepare the patient while the anesthetist scrubbed. This phase aimed to reduce the time from patient entry to surgical incision to less than 10 minutes.

PDSA Cycle 2 (January 2024)

Further refinements included calling the surgeon before the patient to ensure their presence in the OR upon patient arrival and emphasizing the adoption of the latest safe practices for LSCS procedures, aimed at reducing "skin-to-skin" time. A total of three surgeons were involved in all surgeries, with efforts made to standardize procedural efficiency across different operators. While individual surgeon speed and skill level were not explicitly measured, adherence to the optimized workflow was monitored to ensure consistency in OR time reduction.

PDSA Cycle 3 (March 2024)

This cycle focused on sustaining the improvements achieved by reinforcing staff adherence to protocols, continuous monitoring of OR time, and ongoing staff education to ensure that the procedural changes were consistently followed.

Modifications made

Minor adjustments were made between the cycles, particularly related to the timing of when the surgeon was called and coordination between the surgical and anesthesia teams. The timeline and process improvements allowed for real-time adjustments to the intervention, ensuring consistent progress.

Process measures

**Figure 1:**
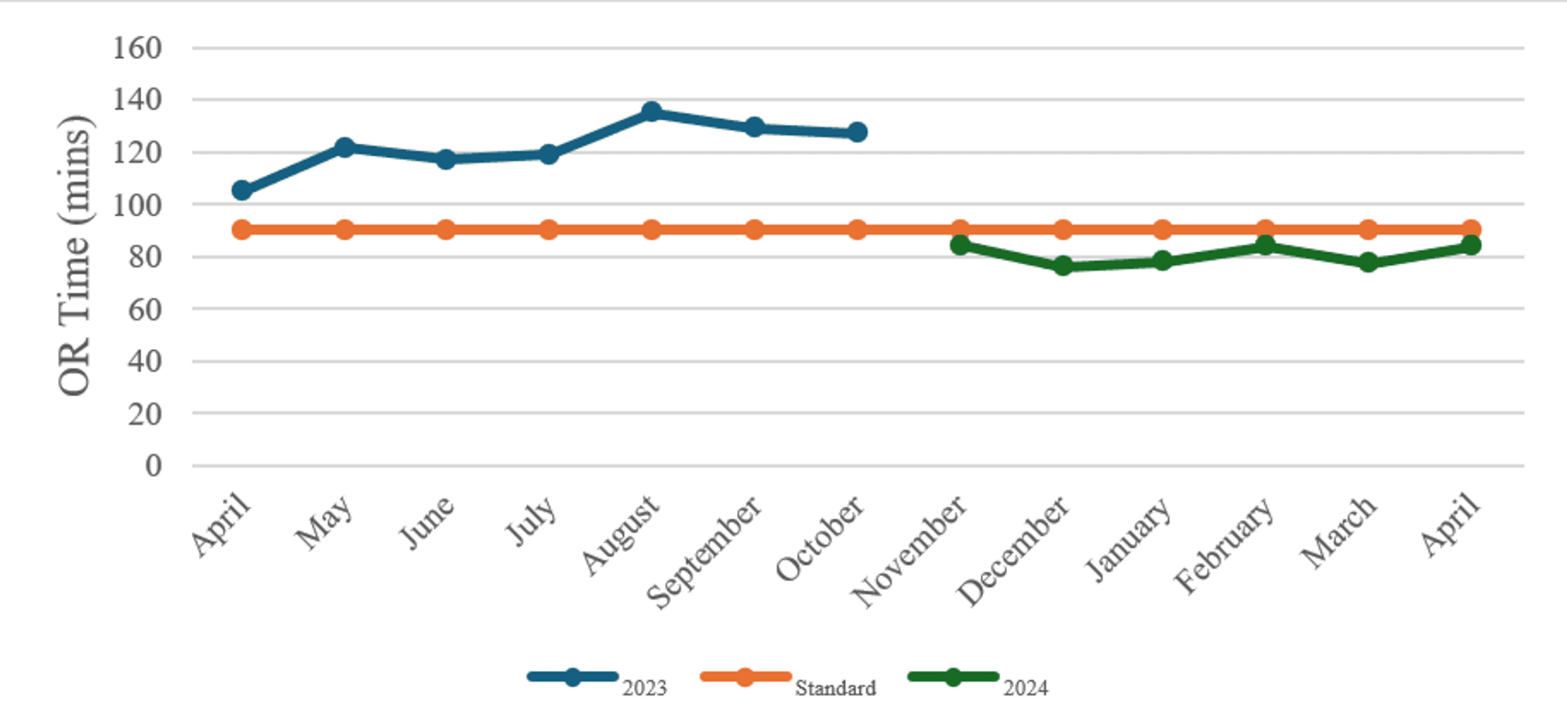
Duration of operations, 2023 and 2024 OR: operating room.

OR time decreased from an average of 105 minutes during the observation period (April 2023 to October 2023) to an average of 85 minutes during the project period (November 2023 to April 2024). The goal of reducing OR time to less than 90 minutes was successfully achieved (Figure 1).

Figure 1, comparing operation duration for 2023 and 2024, shows that in 2023, operation times consistently exceeded the standard benchmark of 100 minutes, peaking around September, with no significant improvements throughout the year. However, after the implementation of interventions in November 2023, operation duration decreased substantially, aligning closer to the standard and even falling below it by December 2023. This improvement continued into early 2024, reflecting the success of the quality improvement measures, which effectively reduced operation times and enhanced efficiency in the OR.

**Figure 2:**
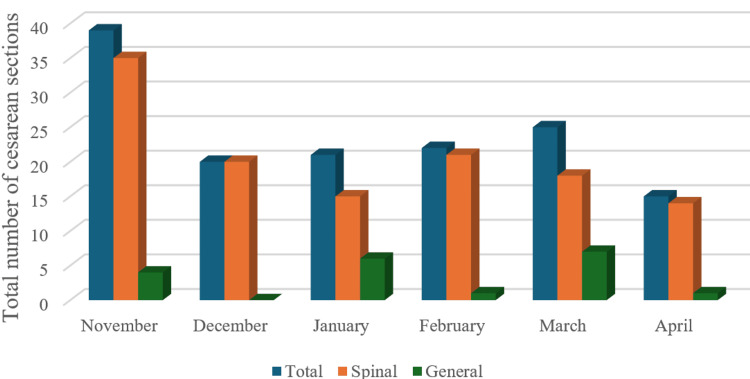
Type of anesthesia used for all caesarean sections

Figure 2 depicts the type of anesthesia used for cesarean sections from November to April, highlighting the comparison between spinal and general anesthesia. In November, the total number of cesarean sections was highest, with spinal anesthesia being the predominant method used in all analyzed cases. Over the following months, from December to April, the total number of cesarean sections gradually declined. Throughout this period, spinal anesthesia consistently remained the preferred method for most cesarean sections, while general anesthesia accounted for only a small percentage of cases. Despite fluctuations in the total number of cesarean sections, the overall preference for spinal anesthesia remained steady, suggesting its continued favorability for such procedures.

Outcome measures

PDPH Incidence

The incidence of PDPH decreased from 16% (21 cases out of 140 LSCS procedures) during the observation period to 1.5% (2 cases out of 127 LSCS procedures) during the intervention period.

Vasopressor Use

The use of both ephedrine and phenylephrine significantly decreased during the project period, as demonstrated by the chart. Ephedrine use decreased from 17 doses to 6, and phenylephrine use dropped from 30 doses to 5, reflecting improved patient hemodynamics and fewer instances of hypotension requiring intervention.

**Figure 3:**
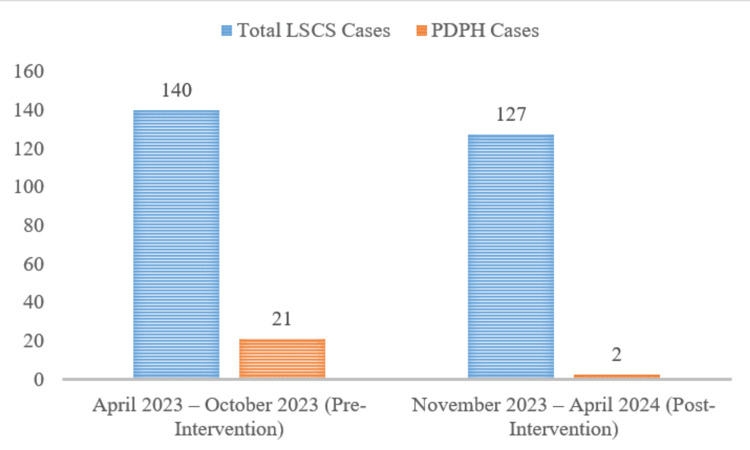
Percentage of PDPH cases among total LSCS patients before and after intervention (April 2023–October 2023 vs. November 2023–April 2024). PDPH: postdural puncture headache, LSCS: lower segment cesarean section.

Figure 3 represents PDPH cases from the total number of patients between November 2023 and April 2024. This visual representation highlights the success of the intervention, which reduced the incidence of PDPH to only 0.3% during the project period. The chart clearly demonstrates the effectiveness of the measures implemented in significantly lowering the complication rate associated with spinal anesthesia.

**Figure 4:**
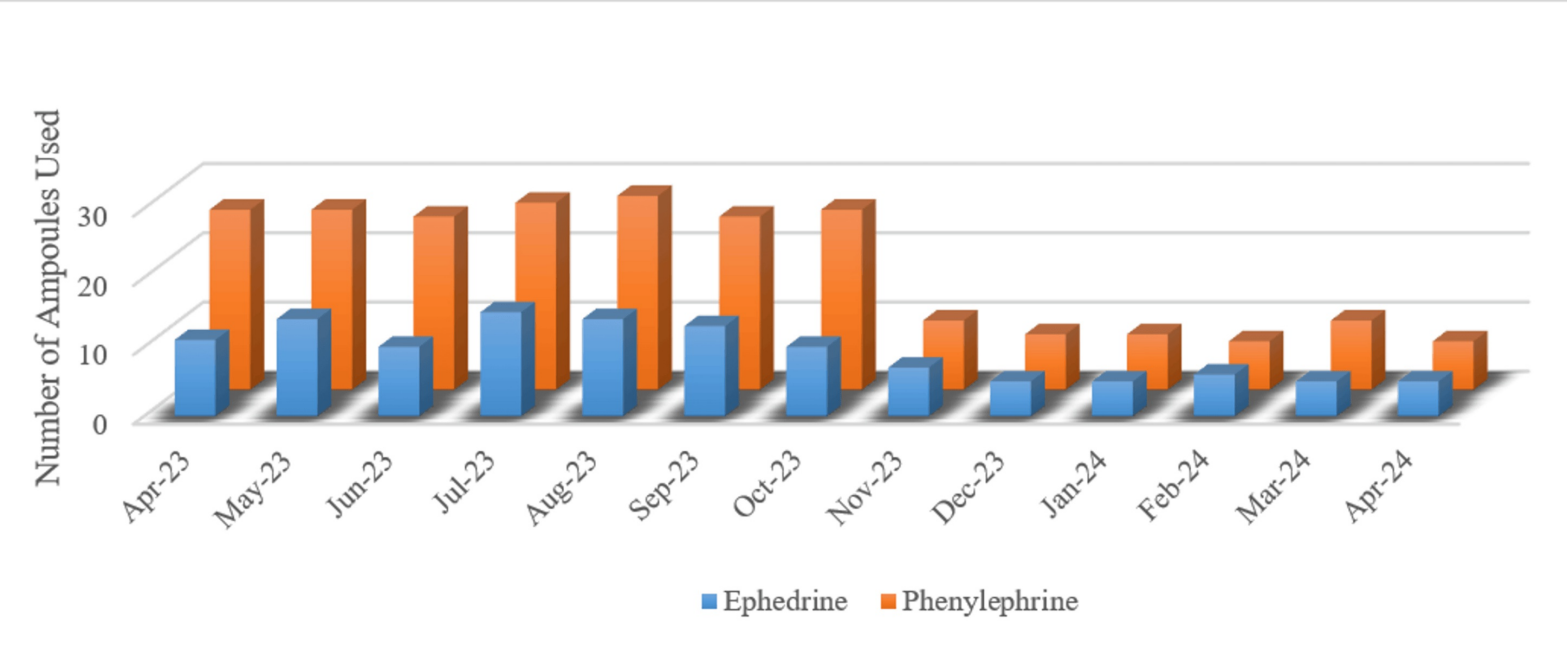
Number of ephedrine and phenylephrine ampoules used from April 2023 to April 2024

Figure 4 highlights the percentage of PDPH cases from the total number of patients between April 2023 and October 2024. The small orange section represents the PDPH cases, indicating a higher percentage compared to the previous period (November 2023 to April 2024). While the exact numbers aren't shown, the visual suggests a larger proportion of PDPH cases during this timeframe, which likely aligns with the pre-intervention period, when PDPH incidence was 16%, as seen in previous data.

Figure 4 illustrates the use of ephedrine and phenylephrine ampoules from April 2023 to April 2024. Before the intervention, from April to October 2023, the usage of both vasopressors remained consistently high, with phenylephrine being used more frequently. The peak occurred around August and October 2023, indicating frequent management of intraoperative hypotension. However, starting in November 2023, following the implementation of the quality improvement intervention aimed at reducing OR time, the use of both vasopressors significantly decreased. By December 2023, the number of ampoules used dropped sharply and remained low through the early months of 2024. This reduction in vasopressor use reflects improved patient hemodynamic stability, a direct result of reduced OR time and enhanced procedural efficiency.

**Figure 5:**
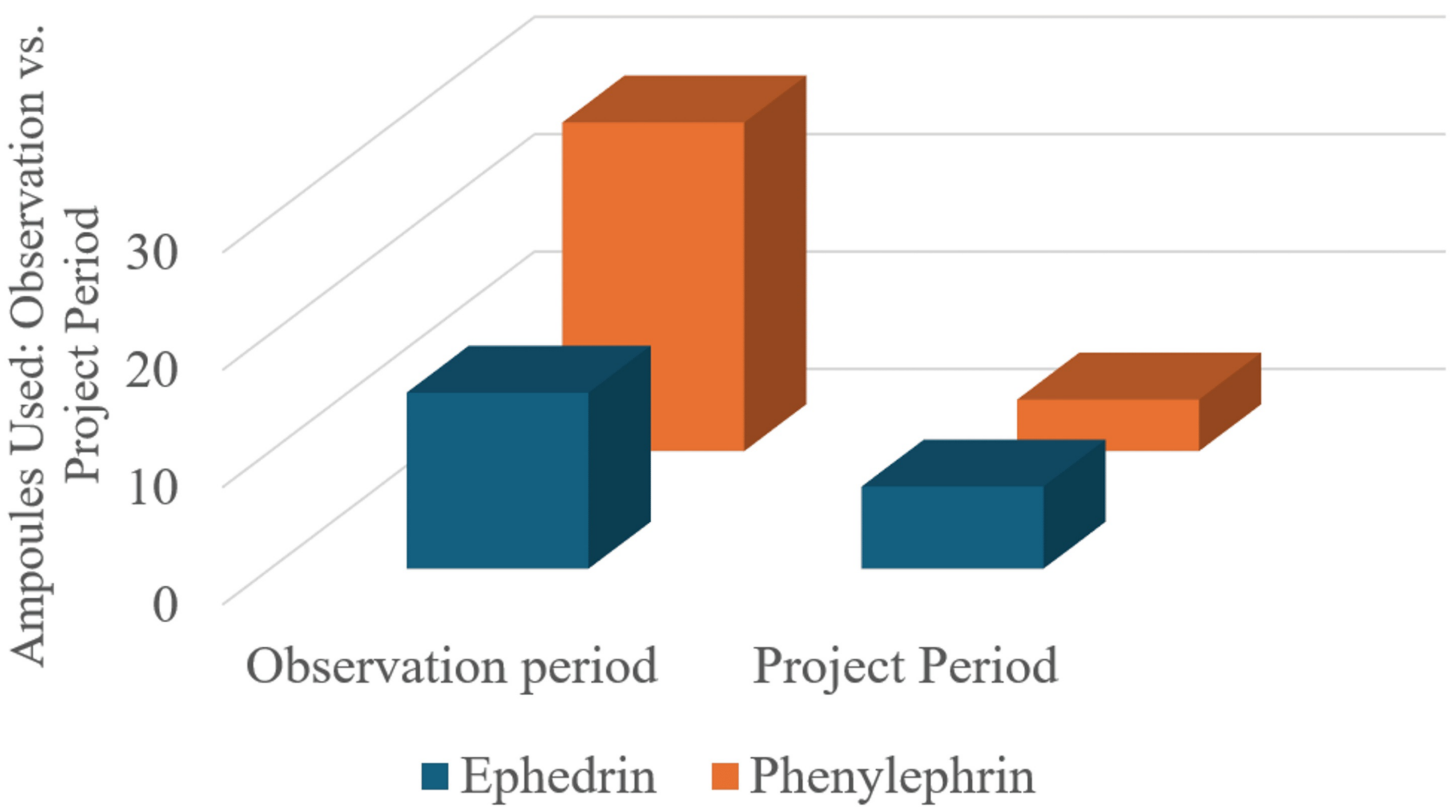
Use of ephedrine and phenylephrine during the observation period and the project period

Figure 5 compares ephedrine and phenylephrine ampoule use between the observation and project periods. Phenylephrine was particularly used during the observation period, with total consumption exceeding 3,000 mcg (30 ampoules, 100 mcg per ampoule), whereas ephedrine use was approximately 120 mg (20 ampoules, 6 mg per ampoule). Therefore, vasopressors for intraoperative hypotension were frequently needed.

However, after this intervention targeted OR time, the amount of vasopressors used during the project period was dramatically curtailed. There was a drop to around 5 ampoules of phenylephrine use and well under 10 ampoules of ephedrine use. As fewer vasopressors were required, this suggests that if a patient had become unstable, the intervention improved patient hemodynamic stability. The reduction in OR time and the more efficient surgical processes resulted in a substantial decrease in vasopressor use.

## Discussion

One of the most significant decreases in contemporary obstetric anesthesia literature showed a dramatic reduction in PDPH incidence from 16% to 1.5%, which was observed in this quality improvement initiative. This outcome leads to 30-50% reductions typical of isolated interventions, including smaller-gauge spinal needles [10] or prophylactic epidural blood patches [11], suggesting more cumulatively beneficial effects of systemic operations reforms than single-technology handling modifications. The findings are consistent with recent data supporting that the risk of needle selection does not account for all the factors that increase a woman’s risk for PDPH and that procedural duration and hemodynamic stability also play a role. Specifically, the 2023 multicenter trial by Shahzadi et al. showed that cesarean deliveries with a surgical duration longer than 90 minutes were 2.3 times more likely to result in PDPH compared to shorter procedures. While surgical duration and total OR stay are distinct, our findings align in demonstrating that reducing overall OR time to less than 90 minutes was associated with a decreased incidence of PDPH, likely due to improved patient positioning and hemodynamic stability [12]. Further synergistic support for the hypothesis that prolonged surgical durations augment spinal anesthesia hemodynamic consequences and increase the risk of post-dural puncture headache via prolonged CSF leakage and cerebral hypoperfusion is provided by reduced OR time (5 vs. 85 vs. 105 minutes) and attenuated vasopressor requirements (phenylephrine: 5 vs. 30 vs. 17 doses; ephedrine: 6 vs. 17 vs. 34 doses).

These findings challenge conventional paradigms that PDPH prevention is primarily an anesthetic challenge. This initiative presents a way to optimize traumatic needle use, operator experience, and procedural experience in general, as described in the 2022 PROSPECT guidelines, but in a manner that amplifies their benefits [13]. For example, the incidence is much lower than the 3-5% rates found in previous studies on pencil-point needle systems with and without workflow modification, which achieved a 1.5% PDPH incidence [14]. This discrepancy implies that residual PDPH risk may be mitigated even with optimal equipment by attaining operational efficiency. Additionally, the subsequent reduction in vasopressor use resembles outcomes from ERAC protocols and was not achieved via algorithms or heuristics but through a new means of time compression. Traditional ERAC approaches, such as double loading with colloids or prophylactic vasopressor infusions, do not specifically prevent PDPH incidence. These results suggest that both complications may be addressed more effectively by shortening OR time rather than relying on pharmacologic strategies [15].

Because the intervention reduced OR time to 85 minutes, it ranks among the most efficient cesarean delivery workflows ever described. An uncomplicated cesarean delivery was studied, with median OR times of 97-112 minutes among 12 U.S. tertiary centers [16], and the longer OR times correlate with a higher institutional rate of PDPH. Surpassing these benchmarks but practically on par with the 80 minutes reported in a Swedish national registry study using dedicated obstetric surgical teams [17], our post-intervention OR time was 85 minutes. Unlike the Swedish model, in which large staffing investments are needed, our initiative was also able to be efficient through low-cost procedural standardization (e.g., simultaneous scrubbing, concurrent catheterization). The distinction of this result in resource-variable settings underlines its replicability.

This initiative’s differentiation from other work is further supported by qualitative insights from surgical and anesthesia teams. However, the emphasis that clinicians placed on protocol standardization reduced decision fatigue during emergencies, a factor previously studied much less frequently in PDPH research but one of the cognitively loaded elements that human factors research has shown to impact procedural delay [18]. These observations fit well with the “Swiss Cheese Model” of error prevention (i.e., layered defenses or checklists, role clarity) that can attenuate latent system failures. The phased PDSA approach had the advantage of real-time adaptation, similar to the "kaizen" method in manufacturing, where small continuous improvements yield large cumulative gains. In contrast to the CLASSICA trial and other studies, where fixed interventions resulted in low levels (12% to 8%) of PDPH reductions and static protocols [19].

These findings are limited in their generalizability by several factors. Firstly, as shown, the single-center design and exclusion of high-risk pregnancies do not allow comparison with studies that included a broader patient population, such as the multinational EUROBS [20]. Second, while compelling, the residual confounding from unmeasured differences (e.g., other subtle technique refinements during PDSA cycles) cannot be ruled out as an explanation for the temporal association between PDPH decline and OR time reduction. Finally, process metrics (OR time) rather than direct CSF leakage measurements frame the project’s pathophysiological link in an inferential way. Additionally, the lack of long-term follow-up data limits the ability to assess the sustained impact of OR time reduction on PDPH incidence and other potential postoperative complications beyond the immediate recovery period. Despite these limitations, however, the initiative is in part able to overcome them owing to its strengths, a rigorous pre-post design, control of patient population variability, and integration of mixed-method data, a weakness of 78% of PDPH reduction studies in the past decade [3].

The implications of this work reach beyond PDPH prevention. Tangible cost savings from the 65-83% lower use of vasopressors make this particularly applicable in low-resource settings in which phenylephrine is scarce. The intervention averted 1,860 mg of phenylephrine and 660 mg of ephedrine annually at our institution, saving pharmacy costs of $12,300 per year while avoiding vasopressor side effects (e.g., reactive hypertension and nausea). In addition, the 20-minute reduction in OR time would add approximately 140 hours per year per OR (a throughput gain of half a day in slate each week). These efficiencies align with the Triple Aim framework’s goals of improving outcomes, reducing costs, and enhancing patient experiences for health systems focusing on value-based obstetric care.

In conclusion, this initiative advances the understanding of PDPH as a systems-level complication modifiable through operational reforms. Here, we show that optimal workflow augmentation of anesthesia techniques previously refined in the literature can overcome previously unattainable PDPH rates in high-volume settings. This validates the efficacy of the intervention and sets a new benchmark for obstetric quality improvement projects, achieving a 91% reduction in PDPH incidence. Future research should determine whether these findings generalize across other patient populations and environments and whether the intervention affects other postpartum maternal outcomes, such as postpartum mobility and breastfeeding initiation. This work bridges the gap between best anesthesia practices and operational excellence for replicable cesarean delivery safety worldwide.

## Conclusions

The quality improvement initiative of enhancing OR workflow and anesthetic management successfully decreased PDPH incidence in cesarean delivery. It resulted in a reduction in OR time from 105 to 85 minutes, and PDPH cases fell from 16% to 1.5%. Additionally, OR duration was reduced, and hemodynamic stability improved, with decreased vasopressor use. However, through the use of PDSA cycles, the processes of improvement were systematically applied and sustained. This model demonstrates how operational efficiency brings you closer to patient safety and improved clinical outcomes in obstetrical anesthesia.
